# Evaluation of AR, AR-V7, and p160 family as biomarkers for prostate cancer: insights into the clinical significance and disease progression

**DOI:** 10.1007/s00432-023-05598-x

**Published:** 2024-02-02

**Authors:** Ruan Pimenta, Feres Camargo Malulf, Poliana Romão, Giovana Vilas Boas Caetano, Karina Serafim da Silva, Vitoria Ghazarian, Gabriel A. dos Santos, Vanessa Guimarães, Iran Amorim Silva, Juliana Alves de Camargo, Saulo Recuero, Bárbara V. Lima Aguiar Melão, Alberto Azoubel Antunes, Miguel Srougi, William Nahas, Katia R. M. Leite, Sabrina T. Reis

**Affiliations:** 1 Laboratório de Investigação Médica 55 (LIM55), Faculdade de Medicina, Hospital das Clínicas HCFMUSP, Universidade de São Paulo, Av. Dr. Arnaldo 455, 2° andar, Sala 2145, Cerqueira Cesar, São Paulo, SP CEP: 01246-903 Brazil; 2 D’Or Institute for Research and Education (ID’Or), São Paulo, SP 04501000 Brazil; 3 Division of Urology, Clinics Hospital, University of São Paulo Medical School, São Paulo, Brazil; 4 Uro-Oncology Group, Urology Department, Institute of Cancer State of São Paulo (ICESP), São Paulo, SP 01246000 Brazil

**Keywords:** Prostate cancer, Androgen receptor, p160, Biomarkers

## Abstract

**Purpose:**

To assess the role of the p160 family, AR, and AR-V7 in different initial presentations of prostate cancer and their association with clinical endpoints related to tumor progression.

**Methods:**

The study sample comprises 155 patients who underwent radical prostatectomy and 11 healthy peripheral zone biopsies as the control group. Gene expression was quantified by qPCR from the tissue specimens. The statistical analysis investigated correlations between gene expression levels, associations with disease presence, and clinicopathological features. Additionally, ROC curves were applied for distinct PCa presentations, and time-to-event analysis was used for clinical endpoints.

**Results:**

The AR-V7 diagnostic performance for any PCa yielded an AUC of 0.77 (*p* < 0.05). For locally advanced PCa, the AR-V7 AUC was 0.65 (*p* < 0.05). Moreover, the metastasis group had a higher expression of SRC-1 than the non-metastatic group (*p* < 0.05), showing a shorter time to metastasis in the over-expressed group (*p* = 0.005). Patients with disease recurrence had super-expression of AR levels (*p* < 0.0005), with a shorter time-to-recurrence in the super-expression group (*p* < 0.0001).

**Conclusion:**

Upregulation of SRC-1 indicates a higher risk of progression to metastatic disease in a shorter period, which warrants further research to be applied as a clinical tool. Additionally, AR may be used as a predictor for PCa recurrence. Furthermore, AR-V7 may be helpful as a diagnostic tool for PCa and locally advanced cancer, comparable with other investigated tools.

**Supplementary Information:**

The online version contains supplementary material available at 10.1007/s00432-023-05598-x.

## Introduction

It is known that Prostate Cancer (PCa) has a significant clinical heterogeneity, ranging from indolent cancers to lethal presentations (Shoag and Barbieri 2016). Thus, the need to predict the progression of the disease for optimal treatment constitutes a modern challenge, and the genomic analysis of the PCa may help us tackle this issue.

Among many genes in this pathology, the Androgenic Receptor (AR) gene has an essential role in PCa (Gulley and Dahut 2002; Assikis and Simons 2004). Many studies have demonstrated the AR function in PCa progression, including the Castration-Resistant Prostate Cancer phenotype (CRPC). Even in low androgen levels, CPRC cells maintain dependence on functional AR (Sharifi 2013; Ferraldeschi et al. 2015; Zhang et al. 2016; Holzbeierlein et al. 2004). Variants of the androgen receptor have also been correlated with CRPC, such as variant seven (AR-V7), which lacks the ligand-binding domain of the wild-type receptor. The variant is hypothesized to be constitutively active and more frequently found in CRPC cells (Hu et al. 2009).

In addition to receptor mutations, countless molecular mechanisms have already been proposed to elucidate this phenomenon. For instance, the change in proportion or expression of AR and its cofactors (coactivators) (Attar et al. 2009). Since androgen cell signaling is essential for the sensitive phenotype (CSPC) transition to CRPC, the numerous AR cofactors might become promising therapeutic targets or PCa biomarkers. However, many of them are unknown (Culig 2016).

The first described coactivators of the AR are the p160 family, composed of three proteins designated as SRC-1 (NCoA1), SRC-2 (TIF2/GRIP1), and SRC-3 (AIB1/NCoA3) (Oñate et al. 1995; Anzick et al. 1997; Chen et al. 1997; Takeshita et al. 1997; Li et al. 1997). SRC-1 is essential for AR activity, especially in hormone-sensitive cells. In vitro*,* models revealed that SRC-1 depletion reduced the proliferation of LNCaP cells (Xu et al. 2009; Agoulnik et al. 2005). Furthermore, it has been correlated with PCa aggressiveness and recurrence (Culig 2016; Agoulnik et al. 2005). Preclinical trials suggest links between SRC-1 and SRC-2 with AR-dependent and independent growth pathways (Agoulnik et al. 2005, 2006). Moreover, the SRC-2 also affects the androgen gene response (Xu et al. 2009) due to interactions in the DNA-binding domain of the receptor (Culig 2016). In addition to acting as a coactivator substitute for the other p160 family proteins, SRC-3 expression has been associated with poorly differentiated carcinomas (Chung et al. 2007; Tien et al. 2009). It has also been associated with hormone-sensitive and resistant cancer phenotypes (Sippell et al. 1994; Ma et al. 2011). Although numerous oncogenic features have been described, no definitive study has characterized the coactivator function in the clinical scenario's initiation and transition of phenotypes.

Therefore, a deeper investigation into critical PCa tumorigenesis-related genes may pave the way for consistent biomarkers to forecast PCa risk, significant PCa, and oncologic outcomes after surgical treatment. According to Sharma P. et al., the use of biomarkers in PCa has evolved significantly, although further research is still required for clinical applications (Sharma et al. 2016). There is already data in the literature associating oncologic outcomes with our genes of interest. Antonarakis ES and colleagues qualitatively demonstrated AR-V7 expression in patients with resistance to hormone therapy and shorter time to biochemical recurrence (Antonarakis et al. 2014). Furthermore, Linja MJ et al. analyzed the expression of the p160 family, among other genes, in clinical specimens. Despite no associations with SRC-2 and SRC-3, the authors reported an inverse relation between CRPC samples and SRC1 expression (Linja et al. 2004). However, other trials reported the association of hormone-resistance PCa and tumor relapse specimens with increased levels of SRC-1 and SRC-2, respectively (Gregory et al. 2001; Culig et al. 2004).

Due to the necessity of better comprehension of AR signaling and its coregulators, the present study aims to evaluate associations between the genetic profile of the p160 family, the AR, and the variant AR-V7 with clinicopathologic features, biochemical recurrence, and CRPC progression in PCa patients who underwent radical prostatectomy.

## Methods

### Patients and ethics statement

The study surgical specimens were secured from 155 treatment-naive PCa patients who underwent radical prostatectomy, composing the intervention group. The surgeries occurred between January 1994 and December 2012 and were performed by a single surgeon (MS). Medical records from his clinic were retrieved to obtain patients’ clinical data. The mean age was 63 years, and the mean follow-up was 84 months (Table 1). The control population comprised the non-cancerous peripheral zone tissue from 26 patients who had a screening prostate biopsy. It is worth emphasizing that the collected pathology specimens from both groups were classified by a uropathologist expert (KRML). The sample was further categorized according to clinical and pathological data. Therefore, subsets were based on PSA levels (divided into < 10 and ≥ 10 ng/ml), pathological staging (divided into pT2 and pT3, which is an organ-confined disease and a non-organ-confined disease, respectively), Gleason’s Score (divided into scores < 7, 7, and > 7), ISUP grading (grades 1 through 5), CRPC phenotype progression (patients who had had a recurrence, then used hormone therapy, and recurred again), biochemical recurrence cancer risk (low-risk disease; intermediate-risk disease; high-risk disease), locally advanced PCa (localized PCa group vs. locally advanced PCa group), metastatic progression, and presence of biochemical recurrence. For better clarification, the division according to biochemical recurrence cancer risk and locally advanced PCa was based on the EAU guidelines, which provide definitions for both classifications (EAU Guidelines 2022). Additionally, the biochemical recurrence definition was based on a post-operative PSA value ≥ 0.2 ng/mL (Paller et al. 2013). The mean value was used for continuous variables as the cutoff for subgroup definition, such as the mean prostatic volume. However, based on the EAU guidelines, the PSA cutoff was set at 10 ng/ml to differentiate a low-risk PSA from a more clinically significant cancer.

**Table 1:** Demographic Characteristics

	PCa Group (N = 153)	Control Group (N = 26)
Age (years)		
Range	38–80	55–73
Mean (SD)	63 (7.93)	64.76 (4.64)
PSA (ng/mL)		
Range	1.50–117	2.2–5.6
Mean (SD)	11.03 (12.53)	4.22 (1.30)
< 10 N (%)	109 (72.2)	6 (100)
≥ 10 N (%)	42 (27.8)	0 (0)
Recurrency Risk Classification		
Group 1 N (%)	21 (13.7)	
Group 2 N (%)	28 (18.3)	
Group 3 N (%)	104 (68.0)	
Pathological Stage		
pT2 N (%)	69 (45.1)	
pT3 N (%)	84 (54.9)	
Gleason’s Score (prostatectomy)		
Range	5–10	
Mean (SD)	8 (1.02)	
< 7 N (%)	11 (7.2)	
= 7 N (%)	33 (21.6)	
> 7 N (%)	109 (71.2)	
ISUP Group		
Group 1 N (%)	11 (7.2)	
Group 2 N (%)	12 (7.8)	
Group 3 N (%)	20 (13.1)	
Group 4 N (%)	64 (41.8)	
Group 5 N (%)	46 (30.1)	
Biochemical Recurrence		
Yes N (%)	99 (67.8)	
No N (%)	47 (32.2)	
Metastatic Progression		
Yes N (%)	44 (32.4)	
No N (%)	92 (67.6)	
CRPC Phenotype		
Yes N (%)	63 (55.3)	
No N (%)	51 (44.7)	
Follow-up time (months)		
Range	1–213	
Mean (SD)	84 (46.00)	

Moreover, follow-up data from the patients were collected for survival analysis. Time to PCa metastasis, time to PCa biochemical recurrence, and time to CRPC phenotype development were obtained. For this analysis, the median of the gene expression levels was applied to separate the groups in “under” or “super-expressed.”

The study was approved under protocol Nº 6.018.973 by the Hospital das Clinicas of the University of Sao Paulo Medical School (HCFMUSP) ethics and the local research committee. All study participants signed an informed consent authorizing the use of surgical specimens.

### Extraction of RNA and quantitative real-time polymerase chain reaction

RNA extraction from the radical prostatectomy sample or the peripheral zone biopsy was performed using the mirVana kit (Ambion, Austin, TX, USA) according to the manufacturer's instructions. The NanoDrop ND-1000 spectrophotometer (Wilmington, DE, USA) was applied to define the concentration of the extracted RNAs. The purity degree was evaluated by the 260/280 nm ratio, using a cutoff of ≥ 1.8 for the selected samples. To assess RNA integrity, agarose gel (0.8%) electrophoresis was performed in three randomly selected samples to check the 28S and 18S bands. Then, the extracted RNAs were stored at – 80 °C until use (Supplement 1). Complementary DNA (cDNA) from the total RNA was generated using a High-Capacity cDNA Reverse Transcription Kit (Applied Biosystems, CA, USA). The target sequence was amplified in a 10 µL reaction mixture containing 2 µL of HOT FIREPol Probe Universal qPCR Mix (Solis BioDyne), 0.5 µL of TaqMan (Supplement 2), and 6.5 µL of nuclease-free water. B2M was used as an endogenous control in the gene expression analysis. The data were analyzed using DataAssist Software (Applied Biosystems, USA). According to a previous study, all qPCR reactions had duplicates (Dos Santos et al. 2022).

### Statistical analysis

The descriptive results were performed using the mean with standard deviation (SD) of gene expression mRNA levels. To compare the expression levels of the genes according to the presence of disease and the clinical characteristics of patients with PCa, we used the Mann–Whitney test, the Student’s T-test, the analysis of variance (ANOVA) test, or the Kruskal–Wallis test. The Shapiro–Wilk test was used for normality analysis. Regarding survival analysis, the Kaplan–Meier method was applied for time-to-event curves. The Log-rank test was used to estimate hazard ratios (HR) for the overall time-to-event comparison between groups. In addition, ROC curves were applied for any PCa, high-risk PCa, and locally advanced PCa to assess the diagnostic performance of the gene expression levels, calculating the area under the curve (AUC). According to Liu X, an optimal cutoff was established for the best-performance gene (Liu 2012). Moreover, Spearman’s test analyzed the correlations between the gene expression levels. Graphics and statistical analysis were performed using GraphPad Prism 9.0 software for Windows, using a significance of p ≤ 0.05.

## Results

### The genetic signature of the p160 family, AR, and AR-V7 in PCa patients and diagnostic accuracy performance

**Fig. 1:**
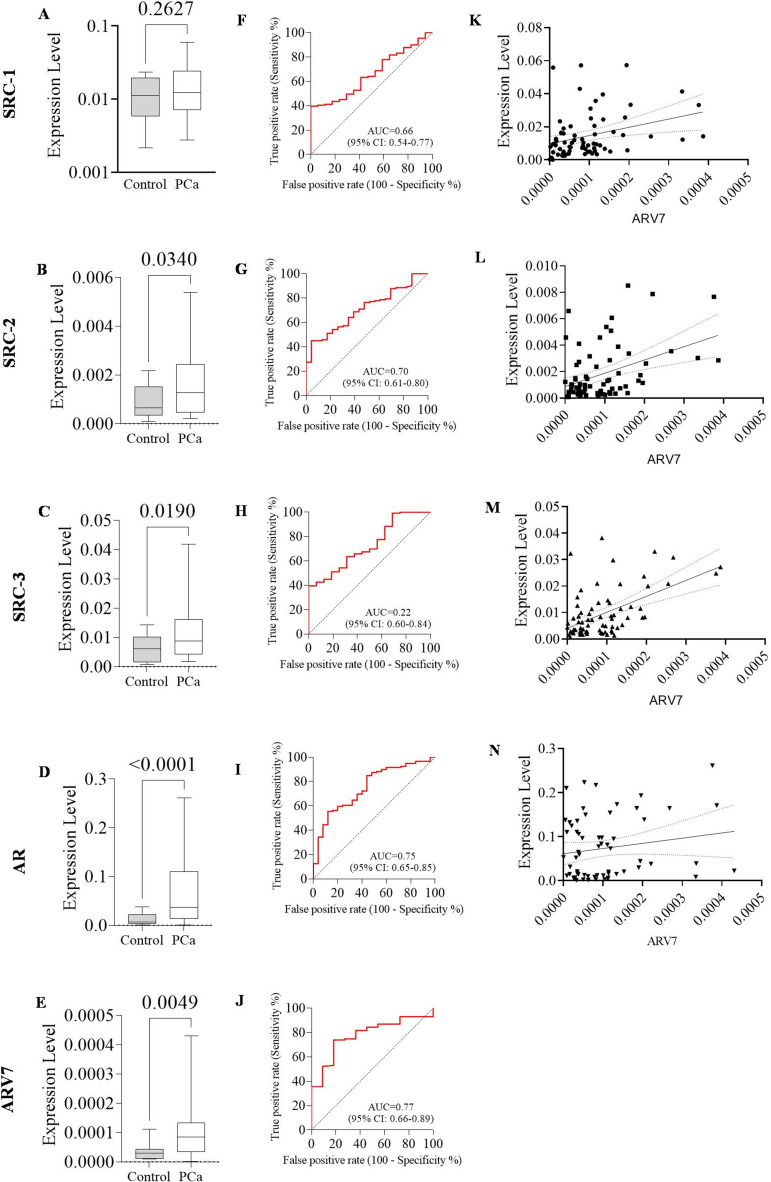
Analysis of each gene according to expression levels in PCa presence; diagnostic performance in any PCa; correlation with AR-V7. **A** Expression level of SRC-1. **B** Expression level of SRC-2. **C** Expression level of SRC-3. **D** Expression level of AR. **E** Expression level of AR-V7. **F** Genetic profile for PCa prediction in SRC-1. **G** Genetic profile for PCa prediction in SRC-2. **H** Genetic profile for PCa prediction in SRC-3. **I** Genetic profile for PCa prediction in AR. **J** Genetic profile for PCa prediction in AR-V7. **K** AR-V7 correlated with SRC-1. **L** AR-V7 correlated with SRC-2. **M** AR-V7 correlated with SRC-3. **N** AR-V7 correlated with AR. The p-values obtained from the statistical analyses are shown above the bars in each panel, and the error bar corresponds to the standard deviation of the samples. T-test was used in all analyses

After qPCR analysis, the genetic profile of PCa patients compared to healthy prostate tissue, the diagnostic performance of PCa, and the correlation among the genes were plotted in Fig. 1. The SRC-2 (*p* < 0.05; Fig. 1B) and SRC-3 (*p* < 0.05; Fig. 1C) genes were super-expressed in the PCa group compared to the control group. The SRC-1 expression in the PCa group was higher than the control, but it did not reach statistical significance (*p* = 0.262; Fig. 1A). The genes AR (*p* < 0.0005; Fig. 1D) and AR-V7 (*p* < 0.005; Fig. 1E) were also super-expressed in the patients with the malignancy when compared with the control group. Regarding the genes with statistical significance, all of them were, at least, expressed more than 1.83 times in the cancer group.

The ability of the genetic profile for PCa prediction was evaluated. In the p160 family, the SRC-1 (AUC 0.66 [95% CI 0.548–0.774]; Fig. 1F), the SRC-2 (AUC 0.70 [95% CI 0.611–0.805]; Fig. 1G), and the SRC-3 (AUC 0.72 [95% CI 0.609–0.845]; Fig. 1H) genes had a significant prediction of PCa. The same occurred for the AR (AUC 0.75 [95% CI 0.655–0.855]; Fig. 1I) and the AR-V7 (AUC 0.77 [95% CI 0.661–0.895]; Fig. 1J) genes. For an AR-V7 optimal cutoff point of 4.930e-005 expression level, the sensitivity and specificity were 73.91% (95% CI 65.21–81.07) and 81.82% (95% CI 52.30–96.77), respectively.

Concerning the AR-V7, it correlated with SRC-1 (Spearman r = 0.358 [95% CI 0.129–0.550]; *p* < 0.005; Fig. 1K), SRC-2 (Spearman r = 0.332 [95% CI 0.101 – 0.528]; *p* < 0.005; Fig. 1L), and SRC-3 (Spearman r = 0.437 [95% CI 0.222–0.612]; *p* < 0.0005; Fig. 1M). Unfortunately, the AR gene expression did not significantly correlate with the p160 family of genes and AR-V7 (Spearman r = 0.127 [95% CI – 0.131 to 0.370]; *p* = 0.31; Fig. 1N). Additionally, the family p160 proteins had significant correlations between them. The SRC-1 gene expression levels correlated with SRC-2 (Spearman r = 0.611 [95% CI 0.459 – 0.729]; *p* < 0.0005; Supplementary material 3A) and SRC-3 (Spearman r = 0.719 [95% CI 0.603–0.805]; *p* < 0.0005; Supplementary material 3A), and SRC-2 also correlated with SRC-3 (Spearman r = 0.778 [95% CI 0.678–0.849]; *p* < 0.005; Supplementary material 3B).

### Association of the genetic profile with clinicopathological features

Concerning biochemical risk group categorization, the high-risk group had 2.44 times higher expression levels than the intermediate group in the SRC-2 gene (*p* < 0.05). Additionally, SRC-3 tended toward super-expression in the high-risk group, with mean expression levels 1.71 times higher than the intermediate group (*p* = 0.063). The remaining genes had no statistically significant associations with the risk groups (Table 2). Only the SRC-2 gene demonstrated a marginal significance for the diagnostic performance of high-risk PCa, with an AUC of 0.59 (95% CI 0.498–0.696; *p* = 0.060; Supplementary Material 4A). After establishing a cutoff 1.46e-3, the sensitivity was 63% (95% CI 53.22–71.82%), and the specificity was 62.22% (95% CI 47.63–74.89%). For clinically locally advanced cancer, SRC-1 (*p* < 0.005), SRC-2 (*p* < 0.0005), SRC-3 (*p* < 0.05), AR (*p* < 0.05), and AR-V7 (*p* < 0.0005) super-expression were significantly associated with locally advanced cancer. The mean expression levels were at least 1.66 times higher than local PCa (Table 2). For the diagnostic performance of locally advanced cancers, AR and AR-V7 demonstrated significant findings. The AR gene had an AUC of 0.64 (95% CI 0.494–0.786; *p* = 0.058; Supplementary Material 4B), whereas the AR-V7 gene had an AUC of 0.65 (95% CI 0.493–0.811; *p* < 0.05; Supplementary Material 4C). The AR-V7 optimal cutoff established at 1.37e-4 revealed a sensitivity of 72.22% (95% CI 49.13–87.50%) and a specificity of 65.26% (95% CI 55.26%–74.08%).

**Table 2:** Description of main results regarding gene expression analysis association with clinicopathological features

Target Gene , mRNA levels	Biochemical Risk Group			
	Low-Risk, Mean (SD)	Intermediate-Risk , Mean (SD)	High-Risk , Mean (SD)	*p*-value
SRC-1	0.012 (0.011)	0.015 (0.013)	0.017 (0.014)	0.370^¶^
SRC-2	0.001 (0.001)	8.18e-4 (7.26e-4)^a^	0.002 (0.002)^a^	**0.012**^**¶**^
SRC-3	0.009 (0.007)	0.007 (0.004)^b^	0.012 (0.011)^b^	0.056^¶^
AR	0.066 (0.080)	0.087 (0.078)	0.067 (0.071)	0.536^¶^
AR-V7	1.13e-4 (9.66e–5)	8.15e–5 (5.82e–5)	1.57e-4 (2.00e–4)	0.243^¶^

Target Gene mRNA levels	Locally Advanced			PSA (ng/mL)		
	No Mean (SD)	Yes Mean (SD)	*p*-value	< 10 Mean (SD)	≥ 10 Mean (SD)	*p*-value
SRC-1	0.015 (0.013)	0.031 (0.032)	**0.0006**^**§**^	0.017 (0.016)	0.017 (0.013)	0.506^*^
SRC-2	0.001 (0.001)	0.005 (0.006)	** < 0.0001**^**§**^	0.002 (0.002)	0.001 (0.001)	0.683^*^
SRC-3	0.009 (0.007)	0.015 (0.014)	**0.016**^**§**^	0.010 (0.009)	0.011 (0.009)	0.920^*^
AR	0.063 (0.064)	0.109 (0.104)	**0.020**^**§**^	0.054 (0.058)	0.106 (0.087)	0.0005^§^
AR-V7	8.35e–5 (5.96e–5)	3.05e–4 (3.48e–4)	** < 0.0001**^**§**^	8.69e–5 (6.61e–5)	2.36e–4 (3.06e–4)	0.032^*^

Target Gene, mRNA levels	Gleason’s Score			
	< 7, Mean (SD)	7, Mean (SD)	≥ 7, Mean (SD)	*p*-value
SRC-1	0.014 (0.015)	0.017 (0.013)	0.015 (0.014)	0.607^∞^
SRC-2	0.002 (0.002)	0.001 (8.79e–4)	0.002 (0.002)	0.563^∞^
SRC-3	0.006 (0.003)	0.008 (0.004)	0.012 (0.011)	0.061^¶^
AR	0.113 (0.102)	0.052 (0.052)	0.064 (0.075)	0.205^¶^
AR-V7	1.43e–4 (1.31e–4)	1.03e–4 (4.91e–5)	0.001 (0.004)	0.108^¶^

Significant *p*-values have been left in bold

*Mann–Whitney

^§^T-test

^∞^Kruskal–Wallis (Dunn’s test)

^¶^ANOVA (Bonferroni correction)

^a^Group 2 vs Group 3: 0.009

^b^Group 2 vs Group 3: 0.022

^c^Group 3 vs Group 4: 0.063

Expression of the whole p160 family was not associated with PSA levels (< 10 or ≥ 10 ng/mL), whereas AR’s mean expression level was 1.96 times higher in the group with PSA ≥ 10 ng/mL (*p* = 0.005) when compared to the group with PSA < 10 ng/ml (Table 2). Also, AR-V7’s mean concentrations were 2.71 times higher in the group with PSA ≥ 10 ng/ml (*p* < 0.05).

No associations were observed between the expression of the genes and the Gleason Score (< 7, 7, > 7) (Table 2). Moreover, categorization into ISUP grading demonstrated significant differences only in Group 5 vs. Group 4 (Table 3). In Group 5, SRC-1 (*p* < 0.05) and SRC-3 (*p* < 0.05) mean concentrations were, respectively, 2.16 and 1.7 times higher than Group 4.

**Table 3:** Description of main results regarding gene expression analysis association with ISUP group, Disease Staging, Metastasis, and Biochemical Recurrence

Target Gene, mRNA levels	ISUP Group					
	1, Mean (SD)	2, Mean (SD)	3, Mean (SD)	4, Mean (SD)	5, Mean (SD)	*p*-value
SRC-1	0.014 (0.015)	0.021 (0.018)	0.018 (0.015)	0.012 (0.009)^a^	0.026 (0.026)^a^	0.022^¶^
SRC-2	0.002 (0.002)	0.001 (8.31e–4)	0.001 (0.001)	0.001 (0.002)	0.002 (0.002)	0.520^¶^
SRC-3	0.006 (0.003)	0.008 (0.004)	0.009 (0.006)	0.010 (0.009)^b^	0.017 (0.016)^b^	0.040^¶^
AR	0.113 (0.102)	0.080 (0.073)	0.036 (0.031)	0.060 (0.065)	0.087 (0.076)	0.084^¶^
AR-V7	6.85e–5 (4.35e–5)	8.51e–5 (3.25e–5)	1.12e–4 (5.92e–5)	8.23e–5 (7.83e–5)	1.53e–4 (1.75e–4)	0.115^¶^

Target Gene, mRNA levels	pT			Metastasis		
	pT2, Mean (SD)	pT3, Mean (SD)	*p*-value	No, Mean (SD)	Yes, Mean (SD)	*p*-value
SRC-1	0.013 (0.009)	0.017 (0.014)	0.064^§^	0.012 (0.010)	0.027 (0.024)	0.002^*^
SRC-2	0.001 (0.001)	0.001 (0.002)	0.772^*^	0.001 (0.002)	0.002 (0.002)	0.388^*^
SRC-3	0.006 (0.004)	0.011 (0.009)	0.048^*^	0.010 (0.008)	0.012 (0.010)	0.321^*^
AR	0.030 (0.031)	0.087 (0.078)	** < 0.0001**^**§**^	0.081 (0.085)	0.085 (0.075)	0.406^*^
AR-V7	8.72e–5 (5.55e–5)	1.29e–4 (1.52e–4)	0.123^§^	8.63e–5 (6.48e–5)	2.40e–4 (3.27e–4)	0.117^*^

Target Gene mRNA levels	Biochemical Recurrence		
	No, Mean (SD)	Yes, Mean (SD)	*p*-value
SRC-1	0.013 (0.007)	0.019 (0.018)	0.103^§^
SRC-2	0.002 (0.002)	0.001 (0.001)	0.070^*^
SRC-3	0.010 (0.007)	0.010 (0.010)	0.534^*^
AR	0.014 (0.011)	0.100 (0.087)	< 0.0001^*^
AR-V7	7.29e–5 (6.27e–5)	1.13e–4 (9.83e–5)	0.0621^*^

Significant *p*-values have been left in bold

*Mann–Whitney

^§^T-test

^∞^Kruskal–Wallis (Dunn’s test)

^¶^ANOVA (Bonferroni correction)

^a^ISUP 4 vs ISUP 5: 0.011

^b^ISUP 4 vs ISUP 5: 0.069

When analyzing pathological staging, the group with non-organ confined disease (pT3) expressed higher levels of SRC-3 (*p* < 0.05) and AR (*p* < 0.0001). Compared to pT2 cancer, the mean levels were 1.83 and 2.9 times higher (Table 3). Although marginally significant, SRC-1 expression levels had 1.3 times higher expression levels in the group with non-organ confined cancer (*p* = 0.064).

With respect to metastatic progression, the group that evolved to metastasis had mean SRC-1 concentrations 2.25 times higher than the subset of patients that did not progress to metastatic disease (*p* < 0.005) (Table 3).

Compared to patients without disease recurrence, patients with recurrence had mean AR levels 7.14 times higher (*p* < 0.0005) (Table 3). Although it had no statistical significance, SRC-2 tended towards super-expression in the non-recurrence group, with mean expression levels 2.0 times higher than the recurrence group (*p* = 0.070). The same occurred with AR-V7 in the recurrence group, with mean expression levels 1.55 times higher than the non-recurrence group (*p* = 0.0621).

**Fig. 2:**
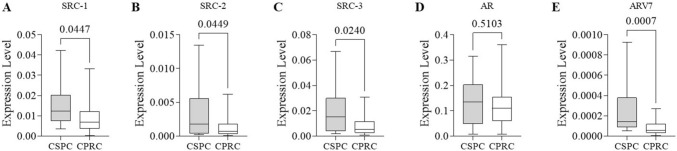
Comparison between expression levels of target genes according to CRPC phenotype. **A** SRC-1 mRNA expression levels in CSPC and CRPC. **B** SRC-2 mRNA expression levels in CSPC and CRPC. **C** SRC-3 mRNA expression levels in CSPC and CRPC. **D** AR mRNA expression levels in CSPC and CRPC. **E** AR-V7 mRNA expression levels in CSPC and CRPC. The p-values obtained from the statistical analyses are shown above the bars in each panel, and the error bar corresponds to the standard deviation of the samples. T-test was used in all analyses

Interestingly, gene super-expression of SRC-1, SRC-2, SRC-3, and AR-V7 (*p* < 0.05 for all except AR-V7—p < 0.005) was observed in the CSPC group. The mean expression levels were at least 1.68 times higher than the CRPC group. AR levels demonstrated no significant differences between the groups (Fig. 2).

### Genetic expression levels in the time-to-event analysis

**Fig. 3:**
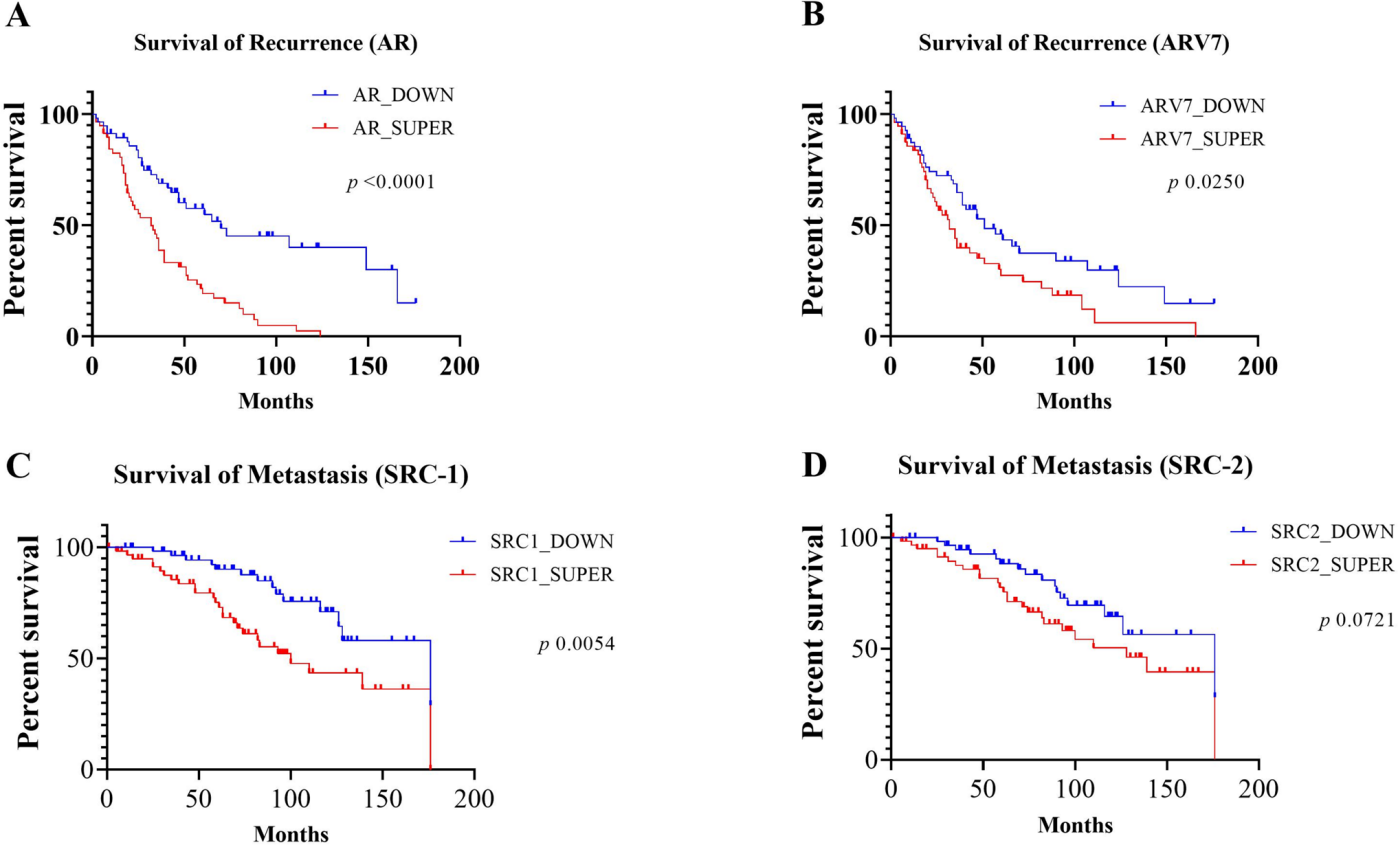
Survival analysis for time-to-recurrence of PCa and for time-to-metastasis of PCa. **A** AR relation with average time-to-recurrence in 32 months vs 70 months. **B** AR-V7 relation with average time-to-recurrence in 32 months vs 51 months. **C** SRC-1 gene relation with average time-to-metastasis. **D** SRC-2 gene relation with average time-to-metastasis. The p-values obtained from the statistical analyses are shown above the bars in each panel, and the error bar corresponds to the standard deviation of the samples. T-test was used in all analyses

Of the 146 patients with follow-up data, 99 had biochemical recurrence (67.8%). Higher expression levels of AR and AR-V7 had a significant association with the overall time-to-recurrence of the disease. In the AR gene, the median time-to-recurrence in the super-expression group was 32 months vs. 70 months in the under-expression group (HR 2.74 [95% CI 1.75–4.28]; *p* < 0.0001; Fig. 3A). Concerning AR-V7, in the super-expression group, median time-to-recurrence was 32 months vs. 51 months in the under-expression group (HR 1.64 [95% CI 1.04–2.58]; *p* = 0.025; Fig. 3B).

Regarding metastasis, 136 patients had follow-up data, with PCa metastasis in 44 patients (32.4%). Super-expression of SRC-1 had a significant association with the overall time-to-metastasis curves. In the SRC-1 gene evaluation, the median time-to-recurrence was 100 months in the super-expression group vs. 170 months in the under-expression group (HR, 2.40 [95% CI 1.28–4.48]; *p* = 0.005; Fig. 3C). Higher expressions of SRC-2 tended to shorter time-to-metastasis, with a median of 128 months vs. 176 months in the under-expression group (HR, 1.74 [95% CI 0.94–3.22]; *p* = 0.072; Fig. 3D).

In addition, for CRPC phenotype analysis, 114 patients had follow-up data, from which 63 developed hormone resistance (55.3%). The gene expression levels were not significantly associated with overall time-to-resistance (Supplementary Material 5 A-D).

## Discussion

One of the main challenges in PCa treatment is the uncertainty of the disease's progression into a significant malignancy. Therefore, appropriate treatment choice is hindered by the overtreatment of indolent cancer and the undertreatment of aggressive disease (Tosoian et al. 2016; Neupane et al. 2021). Several prediction models were developed to tackle this well-known issue, whose final endpoint choice was frequently biochemical recurrence (Neupane et al. 2021). Although commonly applied in these models, clinicopathological variables are not optimal predictors (Zhao et al. 2019). To exemplify, there is considerable evidence that patients with the same Gleason Score can develop different clinical outcomes. Hence, identifying tissue-based molecular markers arises as a possible solution in the scientific community (Zhao et al. 2019; Siadat et al. 2015). Since the androgenic pathway has an established function in PCa cell survival, proliferation, and progression (Obinata et al. 2020), the present study quantified the genetic expression of the following androgen metabolism-related molecules: SRC-1, SRC-2, SRC-3, AR, and AR-V7. The analysis yielded significant associations with the disease’s presence, clinicopathological characteristics, and clinical endpoints.

First, our results demonstrated that all the analyzed genes, except for SRC-1, had a significantly higher expression in patients with PCa, corroborating the importance of the androgenic pathways in PCa tumorigenesis (Aurilio et al. 2020). Even though SRC-1 did not have a statistically significant association with PCa, its mean expression was higher than that of the control group. After evaluation of the genes as a diagnostic tool, AR-V7 performed better than the others, with parameters comparable to recent literature. A systematic revision by Wolf et al. demonstrated that a PSA cutoff of 4.0 ng/mL yielded a sensitivity of 21% with a specificity of 91% for the detection of overall PCa, differently from our study, in which the AR-V7 cutoff point of 4.930e-005 had a sensitivity and specificity of 73.91% and 81.82%, respectively (Wolf et al. 2010). These results also outperformed PSA density (PSAD) as a diagnostic tool for any PCa, with a sensitivity and specificity of 70% for a PSAD cutoff point of 0.15 ng/ml^2^ (Yusim et al. 2020). Despite the AR-V7's comparable performance with the literature, clinical trials comparing them with established biomarkers are required to verify its diagnostic potential.

Surprisingly, our data revealed that the AR gene expression did not correlate significantly with the cofactors and AR-V7 expression. However, the AR-V7 gene had a significant positive correlation with the p160 family protein genes. Current literature demonstrates that the p160 family of coactivators usually binds to the ligand-binding domain (LBD) of the nuclear receptors through LXXLL motifs (McInerney et al. 1998; Darimont et al. 1998; Heinlein and Chang 2002). However, in the AR, it has been demonstrated that the SRC-1 cofactor has a weaker interaction with the LBD and can exert its regulation via interactions in the amino-terminus domain (NTD) (Powell et al. 2004; He et al. 1999; Bevan et al. 1999; Heinlein and Chang 2002). In addition, an in vitro study revealed that peptides that block interactions between SRC-1 and AR also affect the activity of the AR-V7 (Nakka et al. 2013). Therefore, SRC-1 can still induce the androgenic pathway through interactions with an AR variant that lacks the LBD, such as the AR-V7. Interestingly, the strongest correlation was with the SRC-3, which may be explained by its function as a replacement for the other p160 family cofactors (Tien et al. 2009). This concept is further corroborated by Thiyagarajan T. et al., who reported interactions between the cofactor and the NTD of the AR (Thiyagarajan et al. 2023). Moreover, it has already been outlined that the SRC-3 and AR-V7 are associated with the CRPC phenotype. Hence, these findings suggest that the AR-V7 influence on hormone-resistance development depends on the cofactors' activity, especially SRC-3. Functional studies would be appropriate to fully understand this association in the CRPC phenotype development.

High-risk PCa association with the genetic profile did not present with expressive results. All the genes had higher expressions in the high-risk group except for AR. However, a significant difference was only found, in comparison with the intermediate group, for SRC-2 and SRC-3. These results corroborate with the literature, in which SRC-2 is correlated with early disease relapse, a characteristic of high-risk PCa (Gregory et al. 2001; Karantanos et al. 2013). Downregulation of this coactivator decreases the activity of AR-dependent and -independent growth pathways (Agoulnik et al. 2006; Karantanos et al. 2013). In addition, SRC-3 has an essential role in developing poorly differentiated PCa through activation of the PI3K/Akt pathway, which increases the risk of disease recurrence (Xu et al. 2009; Karantanos et al. 2013).

The use of SRC-2 as a diagnostic tool for high-risk PCa reached 63% sensitivity with 62,22% specificity. Despite not being the same parameter, diagnosing significant PCa (defined as ISUP GG ≥ 2) with PSAD demonstrated better performance, with 70% sensitivity and 79% specificity (Wolf et al. 2010). However, another study used a PSA cutoff of 4.0 ng/mL to detect high-grade (defined as GS > 7) PCa, which had a lower sensitivity of 51% (Yusim et al. 2020). Prospective comparative trials are indispensable to truly define the clinical applicability of SRC-2 in diagnosing high-risk PCa.

The patients classified as locally advanced cancer demonstrated higher expression of the target genes, demonstrating the influence of the assessed genes in cancer phenotypes with poorer prognoses, which has already been shown in the literature for SRC-1, SRC-3, and AR-V7 (Xu et al. 2009; Sobhani et al. 2021). Despite the previous ROC analysis of biomarkers to predict clinically significant PCa or high-grade Gleason (Yusim et al. 2020; Wolf et al. 2010), no study assessed a biomarker to predict locally advanced disease. In our research, the best predictor was the AR-V7, which yielded a sensitivity of 72.22% and a specificity of 65,26%. Thus, additional clinical trials will be required to evaluate its predictive potential to distinguish patients with clinically significant cancer who need curative treatment.

It is well established in the literature that AR is directly correlated with both physiological and malignant proliferation as well as the functioning of the prostate (Fujita and Nonomura 2019). Androgens regulate PSA gene expression at the transcriptional level, contributing to a higher PSA serum concentration (Kim and Coetzee 2004). Therefore, it explains our findings on the association of the super-expression of AR and AR-V7 with more elevated PSA concentrations and a worse disease prognosis.

As previously mentioned, the SRC-3 is associated with poorly differentiated PCa, which would be more expressed in the samples with higher Gleason scores. This relation was demonstrated in our study for SRC-3 and SRC-1. Both gene expression levels had the highest concentration in the ISUP 5 group. Still, it was only significantly higher than the ISUP 4 group, probably because most of the sample had this classification. Despite our results, there is no evidence of an association between the increased levels of SRC-1 and cell undifferentiation (Tien et al. 2009). Although recent studies demonstrated that SRC-1 and SRC-3 have different signaling pathways, they share the site of interaction in AR, and SRC-3 can even work as a replacement for the SRC-1 cofactor (Xu et al. 2009; Culig 2016; Tien et al. 2009; Zhou et al. 2010). Therefore, both cofactors may influence poorly differentiated PCa.

The samples with extraprostatic disease demonstrated higher expression of AR and SRC-3. They also tended to higher levels of SRC-1. Indeed, the AR transcriptome plays a pivotal role in regulating cellular metabolism, involving glycolysis, TCA cycle, and FA synthesis, which promotes cell growth and proliferation (Uo et al. 2020). Despite a nonsignificant association, SRC-1 has a biochemical basis for cell invasion and disease progression through increased HER-2 and CSF-1 protein levels (Wang et al. 2009; Tien et al. 2009). Additionally, an in vitro study demonstrated the metastatic potential of breast cancer cells in which SRC-1 regulates cell proliferation and invasion via the SDF-1α–CXCL12 signaling pathway (Xu et al. 2009; Kishimoto et al. 2005). Our metastatic analysis can further corroborate the literature. The higher levels of the SRC-1 gene were associated with higher metastasis incidence and a 2.4-fold increased risk of early metastasis. Hence, SRC-1 is a promising biomarker for metastatic PCa, and deeper investigations are essential to assess it.

Concerning disease recurrence, the AR gene had the most significant association with it. Higher levels of AR were associated with a 73.2% higher risk of earlier recurrence. A similar pattern occurred with the AR-V7 gene, in which the super-expression demonstrated a 62.1% increased risk of earlier recurrence. Even though the literature mainly describes its relationship with the CRPC phenotype progression, our study highlights the importance of androgen signaling in PCa recurrence. Further research will be required to use AR as a clinical tool (Uo et al. 2020).

The main contradictory findings were in the CRPC phenotype analysis. Interestingly, all the genes, except for AR, had higher expression levels in the CSPC group. However, the literature demonstrates an opposite direction, especially for AR-V7, the constitutively active receptor established as one of the mechanisms for developing hormone resistance (Hu et al. 2009). SRC-2 and SRC-3 have been described to be positively associated with the progression to the hormone-resistant phenotype. Androgen blockade increases SRC-2 expression, which activates the PI3K pathway and evolves into metastatic and CRPC presentations (Fujita and Nonomura 2019). SRC-3 was shown to be essential in CRPC development via the induction of Akt and S6K1 expression, with elevated concentrations in CRPC cells (Fujita and Nonomura 2019). It is important to emphasize that our quantitative gene analysis is based on the initial sample, which has not been modulated by hormone therapies and may not show the expression variations described in the literature. We also had a reduced sample size for the CRPC survival analysis, which yielded nonsignificant results. However, we can also hypothesize that the initial PCa samples with lower expression of the androgenic pathway genes suggest that they already depend less on the androgen axis to grow, which is a mechanism for hormone resistance. Although many studies describe the continuous activation of the AR as essential for CRPC progression, some have described independent growth pathways that are active in this phenotype (Hoang et al. 2017). Thus, there is still a need for a better understanding of the mechanisms that drive CRPC. Furthermore, continuous genetic profiling of PCa patients would be interesting to understand how it progresses.

Our main limitations have already been highlighted in the discussion. They are the unbalanced samples for some subgroup analyses (Gleason, recurrence risk classification), the reduced sample size for the control group and the CRPC survival analysis, and the genetic profiling limited to the initial PCa presentations. It is worth mentioning that the classification of high-risk PCa is not established in the literature, and we used one of its definitions, which may affect the analysis if a different one had been applied (Chang et al. 2014). On the other hand, we have to emphasize our approach to PCa oncologic outcome prediction, which is poorly explored in the literature. Rather than analyzing the genetic profile of the sample that had already progressed to the oncologic outcome, we did it in their initial presentation to assess any early changes that could foresee their phenotype progression, which could drive significant clinical applications in the recently operated patients.

## Conclusions

To conclude, the present study demonstrated the importance of AR, AR-V7, and the p160 family of proteins in PCa and its different presentations, giving information to distinguish a clinically significant disease from an indolent one. One of the main findings includes the overt importance of SRC-1 in the metastatic phenotype progression, which warrants further clinical research to be applied as a prognostic factor. In addition, the SRC-2 and SRC-3 cofactors were significantly associated with high-risk PCa, which may aid physicians in distinguishing more aggressive malignancies in the future. The strong correlation between SRC-3 and AR-V7 warrants deeper investigations to establish the paper of the p160 family proteins in the AR-V7 activity for CPRC phenotype development. Regarding the AR gene, its applicability as a diagnostic tool for extraprostatic cancer and as a predictor for PCa recurrence might constitute an effective tool for outcome prediction. Furthermore, AR-V7 was revealed to be a biomarker for PCa and locally advanced cancer, with results comparable to other tools. Therefore, our results described here unveil the potential of the analyzed genes to comprehend the PCa tumorigenesis pathways better and be used clinically as diagnostic or prognostic tools. To confirm these findings, deeper investigation will be warranted, whether in functional, prospective, or head-to-head comparison studies with other established biomarkers.

### Supplementary Information

Below is the link to the electronic supplementary material.

Supplementary file1 (PDF 563 KB)

## Data Availability

The datasets used and/or analyzed during the current study are available to the corresponding author upon reasonable request.
