# Supplementary material for: Evaluation of AR, AR-V7, and p160 family as biomarkers for prostate cancer: insights into the clinical significance and disease progression
Source: J Cancer Res Clin Oncol. 2024 Feb 2;150(2):70. doi: 10.1007/s00432-023-05598-x (PMC10837222; doi:10.1007/s00432-023-05598-x)

Supplement 1. Representative image of the electrophoretic profile of the RNA samples on agarose gel.

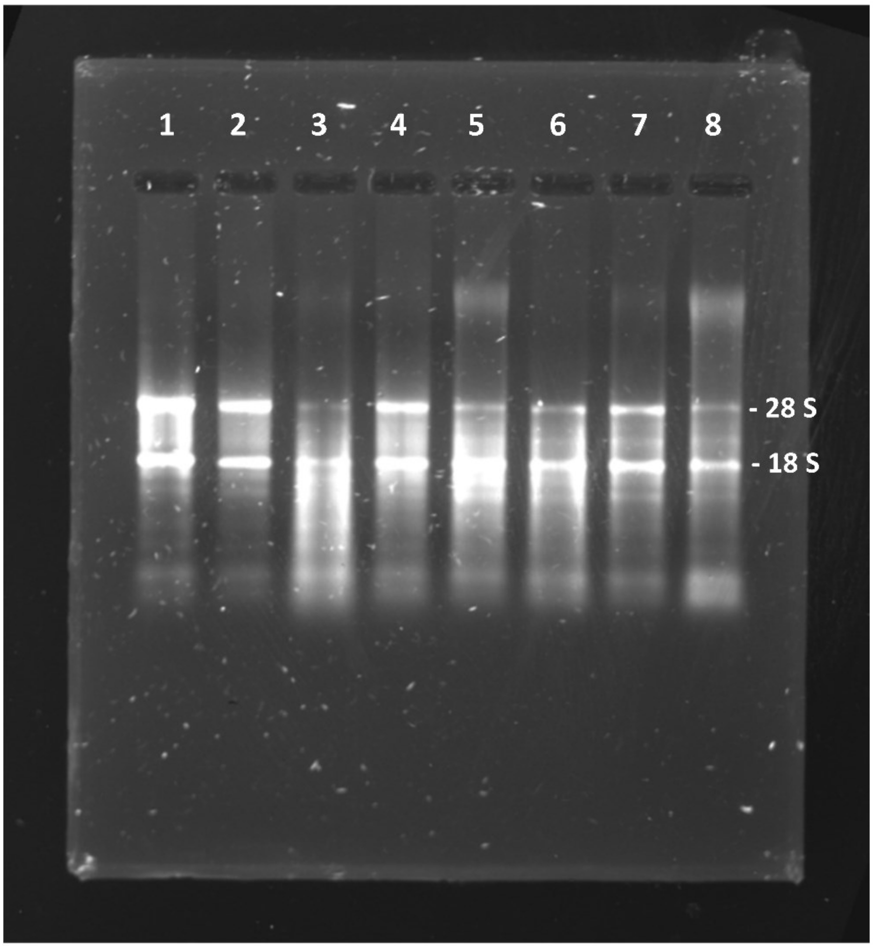

Representative gel electrophoresis image (0.8% agarose, stained with SYBR Safe) of 8 RNA samples extracted with the MirVana kit that represent our sample. The RNA samples were randomly selected, providing an illustrative view of the electrophoretic profile. The 28S and 18S RNAs are represented on the right-hand side of the image for reference.

Supplement 2. TaqMan™ Assay tables were used in the study

Genes	Probes for qPCR (TaqMan)
SRC-1	Hs00186661_m1
SRC-2	Hs00896109_m1
SRC-3	Hs00180722_m1
AR	Hs00171172_m1
AR-V7	Hs04260217_m1
B2M	Hs00187842_m1

Supplement 3. Correlation between p160 family genes.

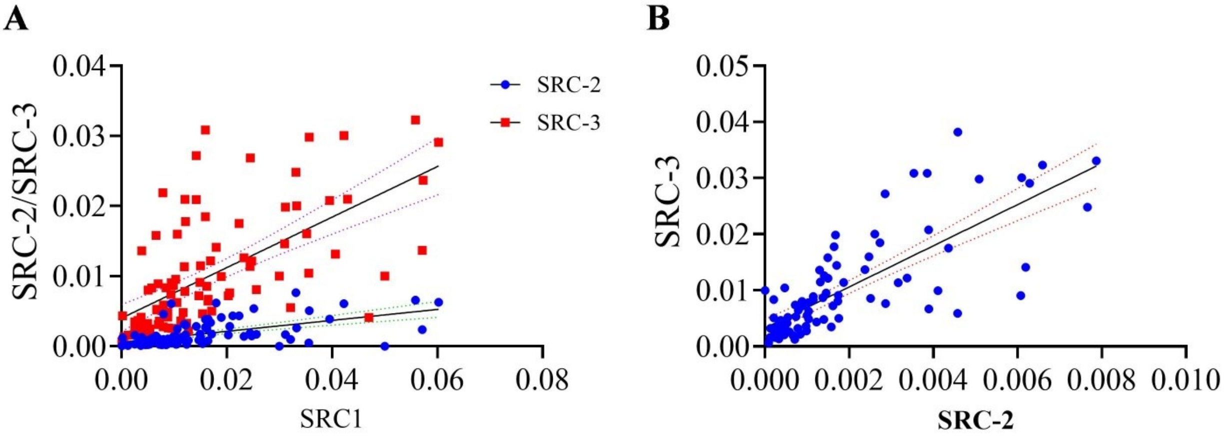

A. Correlation of SRC-1 gene expression concerning SRC-2 and SRC-3 genes (Spearman r = 0.611 [95% CI 0.459 – 0.729]; $p < 0.0005$), (Spearman r = 0.719 [95% CI 0.603 – 0.805]; $p < 0.0005$), respectively. B. Correlation of SRC-2 gene expression with SRC-3 (Spearman r = 0.778 [95% CI 0.678 – 0.849]; $p < 0.005$).

Supplement 4. ROC curves for biochemical risk group categorization.

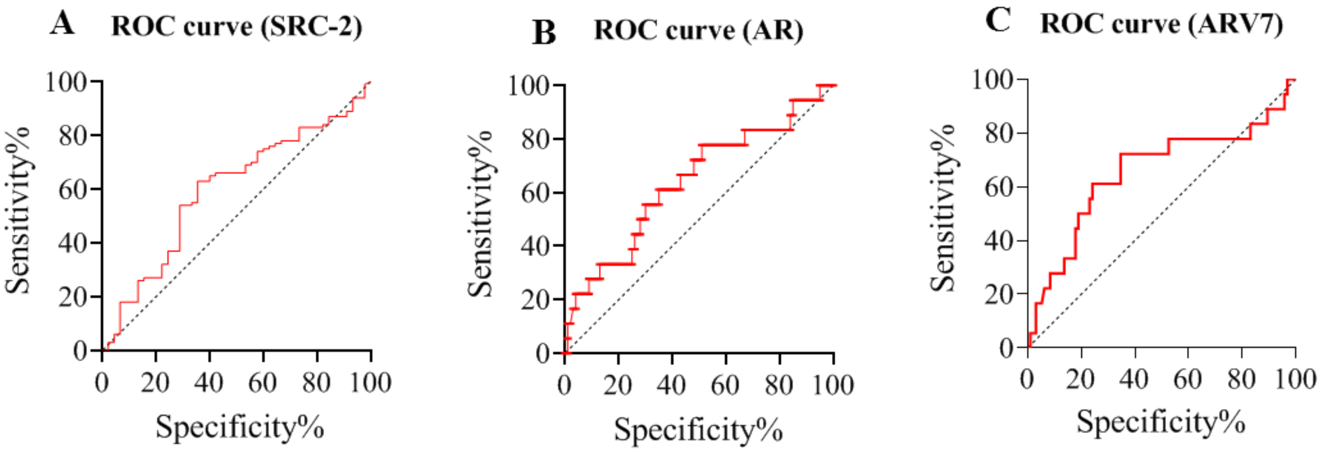

A. Diagnostic performance of high-risk PCa, related to SRC-2 gene [AUC of 0.59 (95% CI 0.498-0.696, p=0.060)]. B. Diagnostic performance for locally advanced PCa, related to AR [AUC of 0.64 (95% CI 0.494 – 0.786; p = 0.058)]. C. Diagnostic performance for locally advanced PCa, related to AR-V7 [AUC of 0.65 (95% CI 0.493 – 0.811; $p < 0.05$)].

Supplement 5. Analysis of the survival rate concerning the expression of the genes of the p160 family.

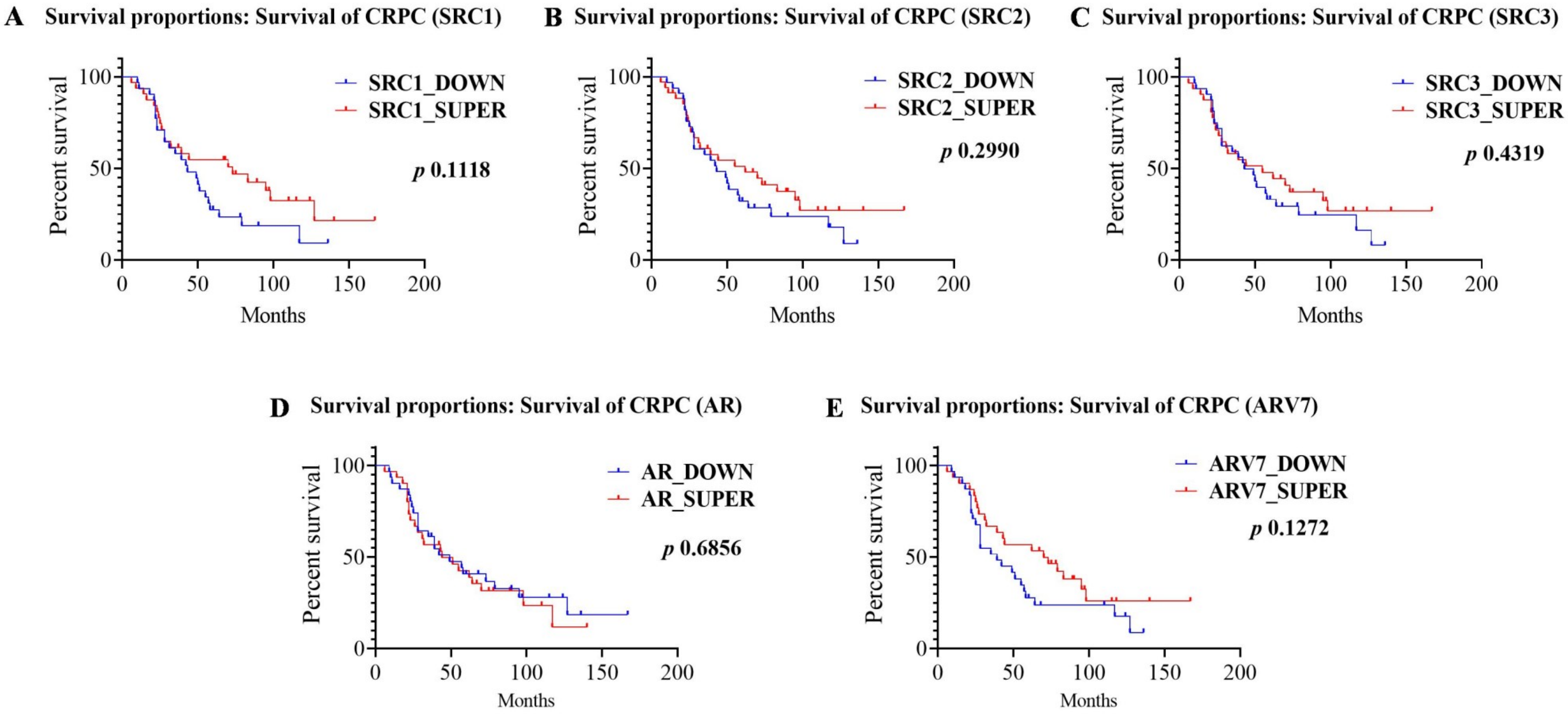

Supplement: Supplementary file 1 — Supplementary file1 (PDF 563 KB) [file 432_2023_5598_MOESM1_ESM.pdf]
